# Lower Functional Connectivity in Vestibular-Limbic Networks in Individuals With Subclinical Agoraphobia

**DOI:** 10.3389/fneur.2019.00874

**Published:** 2019-08-13

**Authors:** Iole Indovina, Allegra Conti, Francesco Lacquaniti, Jeffrey P. Staab, Luca Passamonti, Nicola Toschi

**Affiliations:** ^1^Department of Medicine and Surgery, Saint Camillus International University of Health and Medical Sciences, Rome, Italy; ^2^Laboratory of Neuromotor Physiology, IRCCS Santa Lucia Foundation, Rome, Italy; ^3^Department of Systems Medicine and Centre of Space BioMedicine, University of Rome Tor Vergata, Rome, Italy; ^4^Departments of Psychiatry and Psychology and Otorhinolaryngology–Head and Neck Surgery, Mayo Clinic, Rochester, MN, United States; ^5^Department of Clinical Neurosciences, University of Cambridge, Cambridge, United Kingdom; ^6^Institute of Bioimaging and Molecular Physiology, National Research Council, Milan, Italy; ^7^Department of Biomedicine and Prevention, University of Rome Tor Vergata, Rome, Italy; ^8^Department of Radiology, Athinoula A. Martinos Center for Biomedical Imaging, Boston, MA, United States

**Keywords:** agoraphobia, persistent postural perceptual dizziness, vestibular network, functional connectivity, resting state fMRI, graphs theory

## Abstract

**Background:** Agoraphobia was described in 1871 as a condition of fear-related alterations in spatial orientation and locomotor control triggered by places or situations that might cause a patient to panic and feel trapped. In contemporary nosology, however, this original concept of agoraphobia was split into two diagnostic entities, i.e., the modern anxiety disorder of agoraphobia, consisting solely of phobic/avoidant symptoms in public spaces, and the recently defined vestibular disorder of persistent postural perceptual dizziness (PPPD), characterized by dizziness, and unsteadiness exacerbated by visual motion stimuli. Previous neuroimaging studies found altered brain activity and connectivity in visual-vestibular networks of patients with PPPD vs. healthy controls. Neuroticism and introversion, which pre-dispose to both agoraphobia and PPPD, influenced brain responses to vestibular and visual motion stimuli in patients with PPPD. Similar neuroimaging studies have not been undertaken in patients with agoraphobia in its current definition. Given their shared history and pre-disposing factors, we sought to test the hypotheses that individuals with agoraphobic symptoms have alterations in visual-vestibular networks similar to those of patients with PPPD, and that these alterations are influenced by neuroticism and introversion.

**Methods:** Drawing from the Human Connectome Project (HCP) database, we matched 52 participants with sub-clinical agoraphobia and 52 control subjects without agoraphobic symptoms on 19 demographic and psychological/psychiatric variables. We then employed a graph-theoretical framework to compare resting-state functional magnetic resonance images between groups and evaluated the interactive effects of neuroticism and introversion on the brain signatures of agoraphobia.

**Results:** Individuals with subclinical agoraphobia had lower global clustering, efficiency and transitivity relative to controls. They also had lower connectivity metrics in two brain networks, one positioned to process incoming visual space-motion information, assess threat, and initiate/inhibit behavioral responses (visuospatial-emotional network) and one positioned to control and monitor locomotion (vestibular-navigational network). Introversion interacted with agoraphobic symptoms to lower the connectivity of the visuospatial-emotional network. This contrasted with previous findings describing neuroticism-associated higher connectivity in a narrower visual-spatial-frontal network in patients with PPPD.

**Conclusion:** Functional connectivity was lower in two brain networks in subclinical agoraphobia as compared to healthy controls. These networks integrate visual vestibular and emotional response to guide movement in space.

## Introduction

Agoraphobia (fear of the marketplace), was first described by the German neuroscientist C.F. Westphal in 1871 as a syndrome of altered spatial perception, cognitive distortions about safe locomotion, and fear-driven limitations of mobility (1). Westphal called this condition agoraphobia because he observed it in the busy marketplaces of town squares in nineteenth century European villages. Critically, he considered anxiety, altered spatial perception, and restricted mobility to be “part of one process.” In the modern era, agoraphobia is conceptualized as an anxiety disorder defined solely by fear and avoidance of public places outside of home (2), and the space and motion symptoms that concerned Westphal are no longer part of the disorder. For nearly a century, such symptoms were relegated to the vague notion of psychogenic dizziness, until conditions such as supermarket syndrome (3) and space phobia (4) appeared in the medical literature in the 1970's. These were followed by the syndromes of phobic postural vertigo (5), space-motion phobia (6), and chronic subjective dizziness (7) in which vestibular symptoms and difficulties with exposure to space and motion stimuli were core elements, either co-existing with or pre-disposed and precipitated by anxiety. All of these syndromes provided the background for the recently described vestibular disorder called persistent postural-perceptual dizziness (PPPD), which is defined solely by vestibular symptoms and sensitivity to visual space-motion stimuli (8). Thus, contemporary diagnostic nomenclature (9) separated Westphal's agoraphobia into two conditions, one with anxiety-phobic symptoms and one with vestibular-motion symptoms. This distinction is clinically useful, as agoraphobia and PPPD can be seen quite independently of one another in medical settings, but it should not be interpreted as a formal severing of potential etio-pathogenetic mechanisms. Indeed, Westphal and his contemporaries engaged in lively debates about the relative contributions of visual, vestibular, and psychological processes to the original concept of agoraphobia (10).

Several lines of evidence suggest a shared pre-disposition and partially overlapping clinical manifestations of agoraphobia and PPPD. For example, the personality traits of neuroticism and introversion may be associated with both conditions. A study of twin siblings found independent genetic contributions of neuroticism and introversion to agoraphobia (11). Studies of chronic subjective dizziness found that neurotic and introverted traits were significantly more common in patients with this precursor of PPPD than in patients with other chronic vestibular disorders who had similar levels of dizziness and anxiety or normative samples (12, 13). Studies of PPPD itself also identified levels of neuroticism that were higher than normal (14), and structural vestibular disorders may trigger both PPPD and agoraphobia (8, 15). Investigations of patients with panic disorder and agoraphobia found increased rates of vestibular symptoms, sensitivity to visual motion stimuli, alterations in postural control, and subtle abnormalities in vestibular laboratory tests compared to normal controls (16–21). Conversely, research in patients with the precursors of PPPD found that 60% had anxiety disorders, and in particular panic disorder and agoraphobia (22). Treatment with the selective serotonin reuptake inhibitor (SSRI) paroxetine was shown to normalize changes in postural control in patients with panic disorder (23). Indeed, SSRIs are the mainstay of pharmacological treatment of PPPD, even in patients without psychiatric comorbidity (24).

Given their shared history and the overlapping clinical features, it is possible for agoraphobia and PPPD to share underlying brain mechanisms. Neuroanatomical studies identified extensive connections between vestibular and anxiety systems, extending from the brainstem to the cortex (25), and functional magnetic resonance imaging studies in normal humans revealed significant effects of vestibular stimulation on activity and connectivity in both vestibular and anxiety regions, modulated by neuroticism and introversion (26, 27). Space and motion information in the brain is processed in a widely distributed network, and there is no unimodal primary sensory cortex for vestibular inputs as there are for other sensory modalities. Instead, visual space-motion data are processed by a “multimodal vestibular network” of brain areas that contain neurons that receive combinations of vestibular, visual, and somatosensory stimuli. These multimodal neurons have been found in several regions centered around the parietal opercula, posterior insula, and adjacent posterior perisylvian regions of the parietal and temporal cortex, which constitute the central connectivity nodes of the multimodal vestibular cortex (28, 29) and contribute to perception of gravity (30–32). This network extends to the medial superior temporal area (MST) and posterior inferior temporal gyrus, ventral intraparietal area, superior parietal lobe, somato-motor cortex, hippocampal formation, anterior insula, inferior frontal gyrus, and cingulate cortex (28–32). Some of these multimodal areas (e.g., anterior insula and hippocampus) are critically involved in emotional processing, which may underlie clinical observations of close associations between anxiety and vestibular disorders, including agoraphobia and PPPD (10, 25, 33–37).

The structure and function of brain regions that comprise the multimodal visuo-vestibular network have been studied in patients with PPPD and its precursors using various neuroimaging methods (38–44). In studies that controlled for group-averaged anxiety-related variables but not for their variances, patients with PPPD compared to healthy controls had lower activity, functional connectivity, and cortical folding within the parietal opercula (OP1-4), as well as in a wider network of visuo-vestibular regions including the posterior insula, posterior superior temporal sulcus, superior parietal cortex and motor vestibular regions (38–40). In contrast, patients with PPPD compared to healthy controls had higher fronto-occipital connectivity linked to state and trait anxiety (41, 45). Furthermore, patients with phobic postural vertigo (essentially PPPD plus phobic/avoidance symptoms related to visual space-motion stimuli) had increased connectivity between motor cortex (Broadman area 4—BA4) and orbitofrontal, fronto-polar, and anterior cingulate cortices compared to healthy controls (44).

The brain mechanisms presumed to underlie agoraphobic anxiety have been described extensively (46, 47). In recent neuroimaging studies, patients with panic disorder and agoraphobia showed greater activation than normal controls in the insular cortices bilaterally as well as in the left inferior frontal gyrus, dorsomedial pre-frontal cortex, caudate and hippocampus in response to exposure to symptom-specific pictures compared to healthy controls (48). Activations in the striatum and insula may be stronger in anticipation than actual viewing of agoraphobia-specific stimuli (49). In a non-clinical sample, subclinical agoraphobic symptoms correlated positively with alterations in cortical volumes of the right lingual gyrus, left superior, middle, and inferior temporal gyri, and bilateral calcarine sulci. Exploratory analyses extended those findings to the left pre-central and post-central gyri, the right orbitofrontal cortex, insula, and posterior cingulate gyrus, and bilateral precunei (50). A comparison of these results to those from patients with PPPD suggests potential similarities and differences in task-driven activation (e.g., visual cortical areas—positively correlated with symptom severity in both PPPD and agoraphobia; insula—decreased in PPPD, increased in agoraphobia). However, it is not known if connectivity differs between these disorders. Furthermore, most agoraphobia studies were not controlled for the potential confounds of neuroticism and introversion, which pre-dispose to both conditions (11–14, 45).

In this study we aimed to explore the functional brain connectivity signatures of sub-clinical agoraphobia through a graph-theoretical framework applied to task-free functional MRI in a group of healthy participants selected from the database of the Human Connectome Project (HCP) (51). The extensive information available on subjects in the HCP allowed us to select subjects who reported agoraphobic symptoms and carefully match them to a comparison group with no agoraphobic symptoms on demographics, handedness, and 16 other variables that could interact with agoraphobia such as levels of panic, anxiety, and depressive symptoms, perceived stress, personality traits, negative affect, and self-efficacy. We compared the two groups that we selected from the HCP to test the hypothesis that people with agoraphobic symptoms show lower connectivity in areas of the multimodal vestibular network previously identified in patients with PPPD. In addition, we aimed to assess whether anxiety-related personality traits interact with agoraphobic symptoms to further decrease connectivity.

## Materials and Methods

### Participants

We used rsfMRI data from the S1200 HCP data release, which comprises MRI data and psychological assessments from 1,003 healthy volunteers (http://www.humanconnectome.org/documentation/S1200/). Due to the dominance of the right hemisphere representation of the vestibular function in right-handed individuals (and of the left hemisphere in left-handers) (52) we selected only right-handed individuals, i.e., individuals with handedness score higher than 50 (−100/100 range) (53), to avoid confounds due to different lateralization of functions. Among right-handers, 52 individuals reported agoraphobia symptoms. Experience of at least one episode of agoraphobia, panic or major depression and the number of lifetime depressive symptoms were evaluated by the HCP consortium through the Semi-Structured Assessment for the Genetics of Alcoholism (SSAGA) (54) in agreement with DSM-5 Criteria (2). Anger, aggression, hostility and fear affect were assessed via the NIH Toolbox Fear-Affect Survey, comprising items from the PROMIS Anxiety Item Bank (55). Fear-somatic arousal and sadness were assessed through the Mood and Anxiety Symptom Questionnaire (56, 57) and PROMIS Depression Item Bank (58), respectively. Five Factor Model personality traits were assessed via the NEO five-factor inventory (NEO-FFI) (59). Perceived stress and self-efficacy were scored on the Perceived Stress Scale (60) and on the General Self-Efficacy Scale (http://userpage.fu-berlin.de/~health/selfscal.htm).

### Matching Procedure

**Figure 1:**
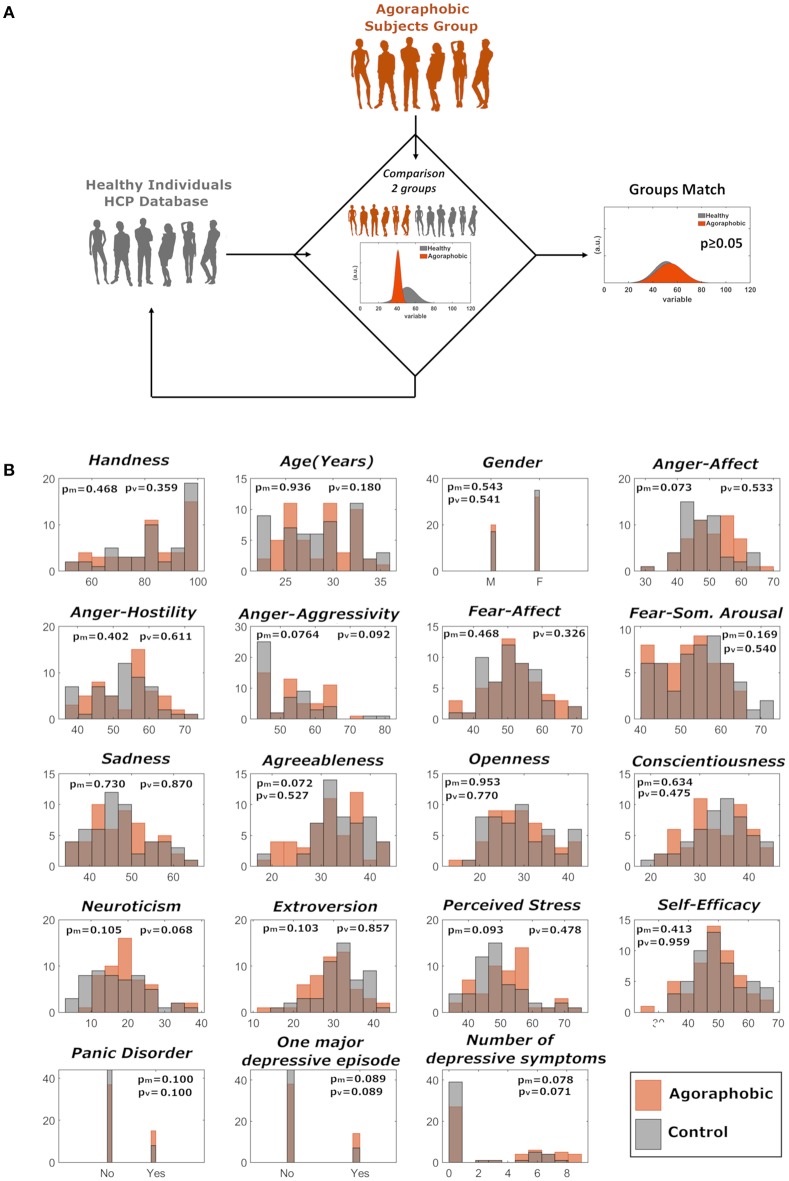
**(A)** Matching of agoraphic and healthy control groups was performed through repeated random sampling (with replacement) of 52 non-agoraphobic subjects and subsequent statistical testing on matching variables. **(B)** Distributions of the demographic, psychological, psychiatric, and personality variables for the two matched groups. Variables relative to agoraphobic and healthy subjects are shown in orange and in gray, respectively. *p*_m_ and *p*_v_ refer to median and variance comparison, respectively.

We matched agoraphobic subjects to 52 healthy right-handed individuals by age, gender, handedness, psychological variables (anger-affect, anger-hostility, anger-aggressivity, fear-affect, fear-somatic arousal, sadness, perceived stress, self-efficacy), presence of psychiatric disorders (panic disorder, one major depressive episode over lifetime, total number of depressive symptoms over lifetime), and personality scores (neuroticism, extraversion, openness, conscientiousness, agreeableness). The matching procedure was as follows: (i) repeated random sampling (with replacement) of 52 non-agoraphobic subjects from the whole (*n* = 1,003 HCP database; (ii) for each sample, group-wise comparison (against the agoraphobia group) of mean values (Mann-Whitney *U*-Test for continuous variables and Chi-Squared test for dichotomous variables) and variances (Brown Forsythe test) for all matching variables, and (iii) acceptance of the first “matched” sample of 52 healthy subjects when all resulting *p*-values (19 comparisons for both means and variances) were *p* ≥ 0.05 (Figure 1).

### Magnetic Resonance Imaging (MRI) Scanning and Definition of Nodes

Within HCP scanning procedures, fMRI data were acquired on a Siemens Skyra 3T in four runs of ~15 min each, through a Gradient-echo EPI sequence (1,200 volume per run, T_R_ = 720 ms; T_E_ = 33.1 ms, FA = 52 deg, FOV = 208 × 180 mm (RO × PE), resolution (x, y, z) = 2.0 × 2.0 × 2.0 mm^3^, Multiband factor = 8, bandwidth = 2290 Hz/Px).

Each 15-min run of each subject's rfMRI data was pre-processed according to Smith et al. (61); it was minimally-pre-processed (62), and had artifacts removed using ICA + FIX (63, 64). ICA + FIX is a data-driven algorithm, which is based on an automated classifier specifically and manually trained to discern, amongst independent component analysis (ICA) results, diverse sources of noise (acquisition, movement, physiological artifacts). The version trained for HPC data has been seen to guarantee an accuracy (as well as sensitivity and specificity) of around 99% (61, 63, 64). Inter-subject registration of cerebral cortex was carried out using areal feature-based alignment and the Multimodal Surface Matching algorithm (“MSMAll”) (65, 66). For feeding into group-PCA, each dataset was then temporally demeaned and had variance normalization applied according to Beckmann and Smith (67), after which group-PCA output was generated by MIGP (MELODIC's Incremental Group-PCA) from 1,003 subjects (68). More details can be found at https://www.humanconnectome.org/study/hcp-young-adult/article/release-s1200-extensively-processed-rfmri-data. While each resulting spatial component map (node map) is obtained through regression and therefore defined over the whole brain, there is a steep roll-off in areas which are mostly related to other components. In order to highlight the brain areas which are dominant/unique in each component, for visualization and description purposes in this paper we thresholded each map at the 98th percentile. For analysis, we employed the individual adjacency matrices computed by the HCP consortium based on group-PCA at dimensionality 100. These which were based on partial correlation coefficients between node timeseries (“netmats2”). The graph representation of each adjacency matrix therefore comprised 100 nodes. Each node can be considered a resting state network in itself [see e.g., (69) for an example at dimensionality 15].

### Graph Theoretical Metrics

After matching, in all adjacency matrices negative correlations were set to zero. For each subject matrix, we calculated node-wise, local graph metrics quantifying the centrality of a node within a network (local strength and betweenness centrality), its ability to transmit information at local level (local efficiency), its integration or segregation properties (clustering coefficient), and its overall influence in a network (Eigenvector centrality) (70). Similarly, for each subject we calculated three overall graph metrics: strength, efficiency and transitivity. These are called *global* graph metrics and provide information about the general topological properties of each subject's connectivity matrix. The first two (strength and efficiency) are calculated by averaging over all nodes, while transitivity is defined for the whole graph. The clustering coefficient of a node estimates how much a single node tends to aggregate, through edges (i.e., connections), with its nearest neighbors. Transitivity is a variant of the clustering coefficient in which a network-wide normalization strategy mitigates the influence of outliers (e.g., of nodes with very low number of connections) on the clustering coefficient. Local efficiency is a similar measure, which estimates, in each location within the graph, how well a node's neighbors can exchange information when the node itself is removed (i.e., it can also be thought of as a measure of resilience). All graph-theoretical measures were computed via the Brain Connectivity Toolbox (70) (https://sites.google.com/site/bctnet/). In addition, given that no consensus exist on thresholding strategies before computing graph-theoretical computations, we repeated the main analyses after thresholding all adjacency matrices at 50% density.

### Statistical Analysis

All metrics were compared between groups (agoraphobia vs. healthy controls) through the non-parametric Mann-Whitney *U* test. Results were corrected for multiple comparisons across the number of nodes (100) using an FDR approach at *p* < 0.05. Whenever a significant group effect was found (*p* < 0.05, FDR-corrected), we separately tested the interaction of agoraphobia and anxiety-related personality traits (neuroticism and introversion) using a general linear model which included group, trait and group^*^trait interaction. Additionally, whenever a significant group effect was found in local graph metrics in a certain node (*p* < 0.05, corrected), we separately ranked the adjacency matrix elements for the agoraphobic and control group in order to identify, for each group, nodes with greatest overall connectivity to the rest of the brain.

## Results

**Figure 2:**
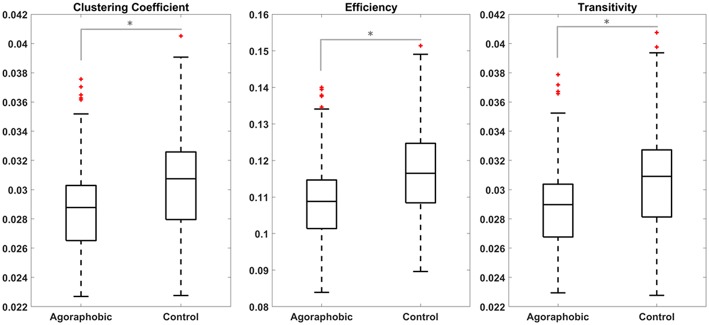
Global clustering coefficient, efficiency, and transitivity were lower in patients with subclinical agoraphobic compared to controls. Figures show the median value (line), quartiles (boxes) and extremes (whiskers) of the three metrics for the agoraphobic and control groups. Comparison between the two subjects groups were performed through non-parametric Mann-Whitney *U* tests.

Globally, we found lower clustering coefficient, efficiency and transitivity in the agoraphobic group compared to the matched control group (*p* = 0.01, 0.01, and 0.02, respectively, Mann-Whitney *U* test) (Figure 2). These results were confirmed when using matrices thresholded at 50% density. In this case, the *p*-values resulting from the Mann-Whitney *U* tests are *p* = 0.014; 0.018; 0.010 for the comparison between global clustering coefficient, efficiency and transitivity of the two groups, respectively. We found two separate networks (ICA components) with significant lower local clustering coefficient and efficiency in subjects with agoraphobic symptoms vs. matched controls. These extended across multiple cortical regions.

**Figure 3:**
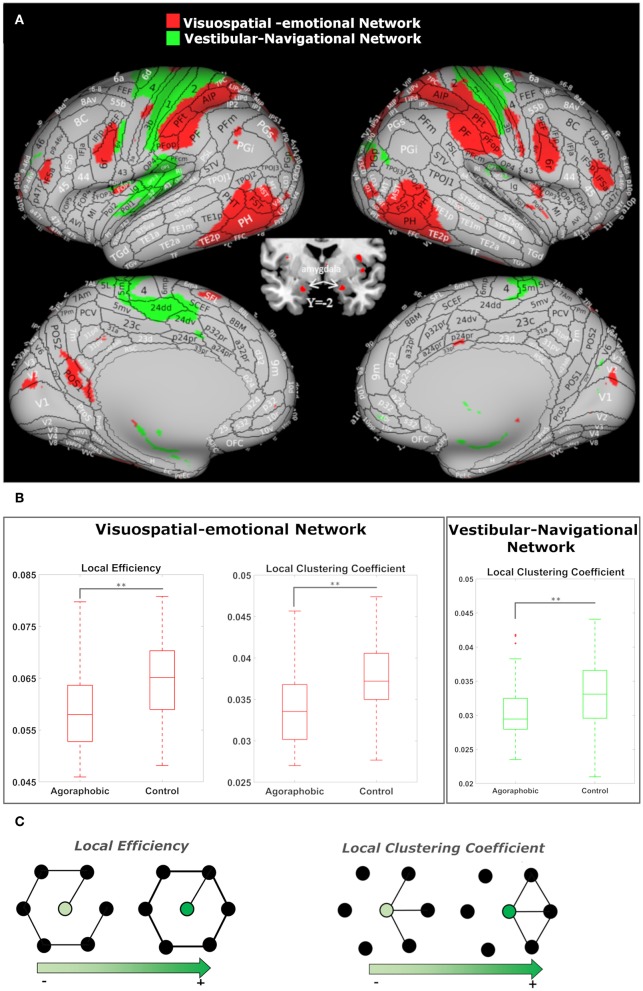
**(A)** The visuospatial-emotional network [node 23, shown in red on the inflated brain (66)] and vestibular-navigational network (node 41, in green) showed reduced clustering coefficients in patients with subclinical agoraphobic symptoms compared to the control group. The visuospatial-emotional network also had a lower efficiency subjects with agoraphobic symptoms. ICAs include values higher than the 98'th percentile. **(B)** The median value (line), quartiles (boxes), and extremes (whiskers) of the local metrics for both nodes are shown. The comparisons between the two subject groups were performed using non-parametric Mann-Whitney *U* tests. *P*-values were corrected for the number of nodes (100) using FDR (*p*-value** < 0.01). **(C)** Examples of networks in which local efficiency and clustering coefficient vary as a function of connections.

The first of these composite networks spanned visual, vestibular, motor, navigation and emotion processing areas (component 23, depicted in red in Figure 3A). The clustering coefficient (*p*_corr_ = 0.0002) and efficiency (*p*_corr_ = 0.0003) of this network were both lower in the agoraphobic than control groups (Figures 3B,C). This component contained a small area at the interface between V1, V2, and MST and extended anteriorly into the ventral visual stream in the fundus of the superior temporal sulcus (FST), basal parietal regions (area parietalis basalis in limine temporali PHT, PH) and posterior area temporalis proper (TE1p, TE2p) [Von Economo nomenclature (71)]. Medially, it encompassed the parieto-occipital sulcus POS1 in the precuneus on the left. Additional parietal regions included the posterior and superior angular gyrus (PGp andPGs) in the caudal inferior-parietal lobe (cIPL); the supramarginal gyrus (PF), PF opercularis (PFop), and tenuicorticalis (PFt) in the rostral IPL and lateral, medial and ventral intra-parietal area (LIP,MIP, and VIP) in the superior parietal lobe (SPL); and area 3a. The posterior insula was included on the right (PoI1-2). This composite network extended into the rostral and ventral parts of area BA 6 (6r and 6v) in the pre-motor cortex and into the frontal operculum (Fop2) and inferior frontal sulcus (IFS) in the pre-frontal cortex. Subcortical structures, specifically the amygdala, head of the caudate nucleus, putamen, and anterior thalamus also were part of the network. We termed this component the “visuospatial-emotional network” because it encompassed brain regions that integrate incoming multi-modality space and motion information (V1/V2, ventral visual stream, cIPL, and posterior insula) (72, 73), areas that process emotions (amygdala and associated subcortical nuclei), and regions that initiate/inhibit movements (pre-motor and pre-frontal cortices).

The second composite network encompassed the left posterior insula, retroinsula, and granular insula, and bilateral parietal operculum (OP1,2,3,4), primary somatosensory and motor cortex (BA 1, 2, 3b, 4), pre-motor cortex (6d), medial SPL, cingulate cortex (5m, 5L, 24), and rostral hippocampus (component 41, depicted in green in Figure 3A). In this network, only the clustering coefficient (*p*_corr_ = 0.0009) was lower in subjects with agoraphobic symptoms compared to controls (Figure 3B). We named this component the “vestibular-navigational network” because it linked the principal vestibular cortical regions and the hippocampus to somatosensory and motor cortices. In summary the visuospatial-emotional and vestibular-navigational networks both extended across parietal, insular and pre-frontal cortices of the brain.

**Figure 4:**
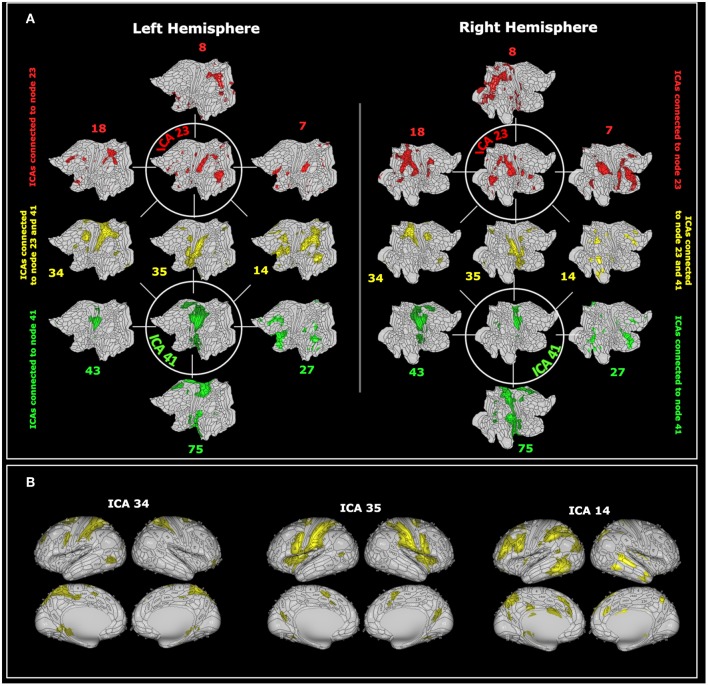
**(A)** The visuospatial-emotional network centered about ICA 23 (in green) and the vestibular-navigational network centered about ICA 41 (in red) are superimposed on flat brain (66). The visuospatial-emotional network was strongly connected with regions 18, 8, 7, whereas the vestibular-navigational network was connected with regions 43, 75, 27. The two networks overlapped at nodes 34, 35, and 14 (in yellow). **(B)** The shared nodes are depicted again superimposed on the inflated brain (66). For visualization purposes we only reported the 6 nodes most closely connected to ICA 23 or 41. ICAs include values higher than the 98'th percentile.

These networks are mostly connected to three common components (in yellow in Figure 4) plus additional components that spread again over the parietal, insular and pre-frontal cortices (Figure 4).

These results were confirmed when using matrices thresholded at 50% density. In this case, individuals with subclinical agoraphobia were found to have lower clustering coefficient (*p*_corr_ = 0.023) and local efficiency (*p*_corr_ = 0.023) in visuospatial-emotional network (node number 23) as compared to controls. In addition, in subclinical agoraphobia the local clustering coefficient was lower (as compared to controls, *p*_corr_ = 0.026) also in the vestibular-navigational network (node 41).

**Figure 5:**
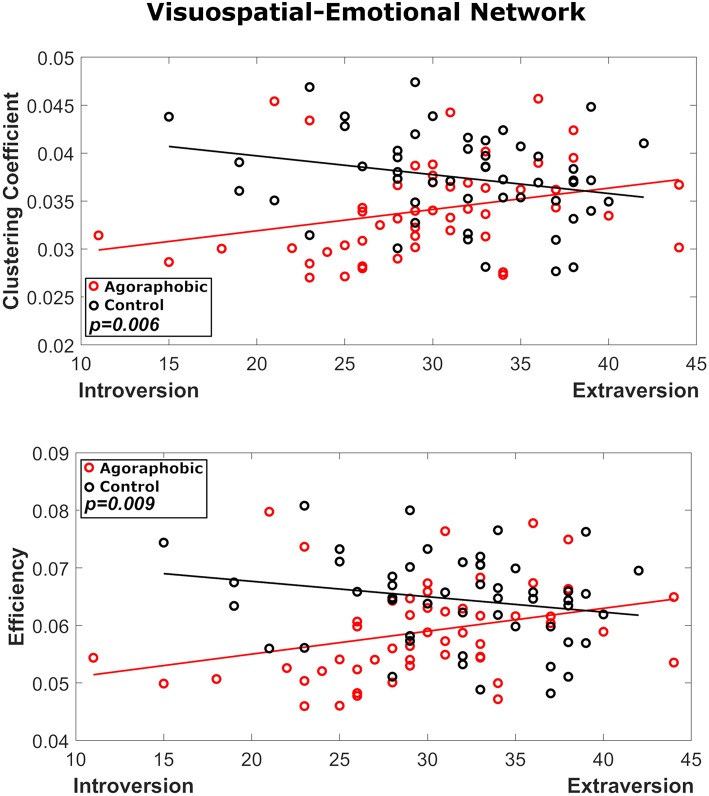
Results of the interaction analysis of agoraphobic group and introversion, where red and black symbols refer to the agoraphobic and control groups, respectively. In node 23 both local efficiency (*p* = 0.009) and clustering coefficient (*p* = 0.006) were significantly affected by the interaction of agoraphobic group and introversion.

We did not find a main effect of introversion (all *p*'s > 0.6) or neuroticism (all *p*'s > 0.1) on graph theoretical-metrics. In the visuospatial-emotional network, however, introversion interacted significantly with the main effect of agoraphobia to reduce the clustering coefficient and efficiency of subjects with agoraphobic symptoms compared to controls (*F* = 7.9, *p* = 0.006 and *F* = 7.0, *p* = 0.009, respectively) (Figure 5). Thus, individuals with agoraphobic symptoms and introverted personality traits had the lowest connectivity metrics. Introversion did not interact significantly with agoraphobia in the vestibular-navigational network. Neuroticism had no effect on either network.

These results were confirmed when using matrices thresholded at 50% density. Also in this case, in the visuospatial-emotional network both local efficiency (*p* = 0.012) and clustering coefficient (*p* = 0.009) were significantly affected by the interaction of agoraphobic group and introversion.

## Discussion

In this study, we were able to utilize the detailed information of the HCP database to identify a group of 52 subjects with sub-clinical agoraphobic symptoms and match them closely to a control group of 52 subjects across a large number of potential confounds. Critically, matching was done not only for mean values of potentially confounding variables, but also for their variances. These strict criteria enabled us to study the neural correlates of agoraphobic symptoms far more independently of confounding influences than previous studies of agoraphobia and PPPD. In the best controlled investigations of PPPD published to date, for example, patient and control groups were matched for mean scores of measures of state anxiety and depression and five personality factors (39, 40, 45). We also were able to exploit the image quality of the HCP database to extract a high granularity yet robust decomposition of brain nodes and networks through group-ICA (dimensionality 100).

First, we found that agoraphobic symptoms were related to lower global efficiency, clustering coefficient and transitivity, reflecting overall lower integrative functioning across the entire brain. Second, through analyses of local, component-wise effects, we identified two networks which, when comparing patients with agoraphobic symptoms to healthy controls, scored lower in at least one connectivity measure. The visuospatial-emotional network included portions of the primary visual cortex, ventral visual stream, multiple regions of the parietal lobe, and parts of the pre-motor and pre-frontal cortices as well as the anterior thalamus, basal ganglia, and amygdala. This network would be well-positioned to process incoming visual stimuli (occipital lobe and visual stream), link them to vestibular and somatosensory inputs (parietal association areas and posterior insula), and use this information to plan and initiate or inhibit locomotor commands (pre-motor and pre-frontal regions) in response to desires and threats in the environment (amygdala and associated subcortical structures). However, the lower level of efficiency and clustering of this network suggests that patients with sub-clinical agoraphobic symptoms may not incorporate all of this information into high level management of their behaviors, but perhaps react more instinctively to agoraphobic stimuli. The fact that introversion further reduced efficiency and clustering of this network raises the possibility that individuals with this personality trait are even more strongly driven by innately determined reactions to agoraphobic stimuli.

The vestibular-navigational network encompassed core regions of the multimodal vestibular cortex in the parietal operculum bilaterally plus areas of the primary somatosensory cortex, motor cortex, pre-motor cortex, cingulate and hippocampus. This suggests a pre-dominant role on the output side of locomotor control. The vestibular-navigational network is well-positioned to control (motor and pre-motor cortices) and monitor (somatosensory and vestibular cortices) movements in space (hippocampus) and compare intended to actual outcomes (cingulate cortex). The lower level of clustering of this network corroborates the fact that agoraphobic stimuli are salient for networks that are dedicated to control of movement in space.

Spatial and navigation systems are characterized by a high degree of redundancy. Detection of movement of self is supported by the multimodal nature of the vestibular cortex in which vestibular signals, somatosensory inputs, and optic flow all provide information about self-motion (74). Detection of movement in the environment is much more dependent on visual information (hearing plays a lesser role in humans), but vestibular and somatosensory inputs are necessary to stabilize the eyes on targets of interest. Navigation is supported by neurons that are sensitive to the direction and speed of self-motion. These are redundantly located not only in the hippocampal formation and entorhinal cortex, but also across the parietal cortex and subcortical structures (73). Thus, reductions in integrative functioning of the visuospatial-emotional and vestibular-navigational networks in individuals with self-reported agoraphobic symptoms does not preclude adequate processing of visual space-motion data or acceptable control of movement but indicates that these networks may weigh data that they process differently than normal. One example is visual dependency, i.e., the tendency to rely more strongly on visual than vestibular or somatosensory information for spatial orientation. This over-reliance on visual inputs has been reported in patients with agoraphobia, perhaps because vision can detect threats at a distance whereas vestibular and somatosensory systems require contact with the body. Regardless, over-weighting of visual information may cause spatial disorientation in environments with complex patterns or multiple moving objects (75).

The visuospatial-emotional and vestibular-navigational networks identified in patients with sub-clinical agoraphobia have comparable, though not identical, counterparts in patients with PPPD. Studies using sound-evoked vestibular stimuli found reduced activity and connectivity in a network encompassing the parietal opercula, posterior insula, posterior superior temporal sulcus, superior parietal cortex and motor vestibular regions in patients with chronic subjective dizziness, a PPPD precursor, compared to healthy controls (38). This is roughly analogous to the vestibular-navigational network identified in this study of patients with agoraphobic symptoms. In addition to the overlapping brain structures that comprised these networks, neither was influenced by neuroticism or introversion. Investigations employing visual motion stimuli in patients with PPPD identified a link between visual (V3 and middle occipital gyrus) and frontal regions (inferior frontal gyrus/anterior insula) (40), but this was more limited than the visuospatial-emotional network found in patients with agoraphobic symptoms in that it did not extend into the parietal lobe, posterior insula or pre-motor areas. Furthermore, neuroticism was associated with increased connectivity in the visual-frontal network of patients with PPPD (45) whereas introversion interacted with agoraphobic symptoms to decrease connectivity in the visuospatial-emotional network of patients with subclinical agoraphobia. Interestingly, neuroimaging findings in a study of patients with phobic postural vertigo (i.e., individuals with PPPD plus substantial phobic/avoidant symptoms) were centered about vestibular cortical regions of the parietal operculum and insula, but also extended into the motor cortex, orbitofrontal, and anterior cingulate cortices (44). Those results were not adjusted for state anxiety though half of the patients were too anxious to tolerate the confined spaces of the MRI scanner, suggesting that the associated phobic avoidant symptoms rather than core elements of PPPD were responsible for the broad changes in brain structure and function.

Taken together with previous neuroimaging work on PPPD and its precursors (38–45), the results of this investigation provide information about the architecture of brain networks subserving spatial orientation, control of locomotion, and threat assessment as they relate to PPPD and agoraphobic symptoms. They indicate that Westphal's observation that one process linked alterations in sense of space, control of movement in motion-rich environments, and instinctive fear reactions (1) was not an error but may involve two linked networks in the brain. At the same time, they also suggest that Westphal's agoraphobia had two components that reflect the contemporary separation of agoraphobia and PPPD into separate clinical entities. The first component involves the detection and processing of afferent visual space-motion information and any threats contained therein, plus initiation or inhibition of behavioral responses. The brain network underlying these functions is less well-connected in patients with agoraphobic symptoms than in normal individuals, an effect that is amplified by the severity of introversion. In contrast, an overlapping, but narrower network of visual-frontal regions shows greater connectivity in patients with PPPD compared to normal controls in proportion to the severity of neuroticism (45). With regard to this component, patients with phobic postural vertigo (i.e., PPPD plus phobic/avoidance symptoms) seem to possess alterations in brain functioning that are closer to agoraphobia than PPPD alone (44). The second component involves the motor control and monitoring of locomotion. The brain network underlying these functions is similar in patients with subclinical agoraphobia and PPPD. It is less well-connected in both disorders, but not affected by anxiety-related personality traits in either one.

## Limitations of the Study

The investigation of a cohort of individuals with subclinical agoraphobic symptoms may be considered a strength and a weakness of study design. On the positive side, we were able to study the neural correlates of visual space-motion processing in our subjects without the potential confounds of serious panic/phobic symptoms. On the negative side, our findings are limited to sub-threshold symptoms and may not apply to patients with fully developed diagnoses of agoraphobia. This limitation is mitigated by the observation of other investigators that agoraphobic symptoms lie on a continuum from normal to pathological levels (50); that is, patients with the clinical diagnosis of agoraphobia have quantitatively, not qualitatively, different symptoms than individuals with subclinical presentations. Thus, we likely investigated one end of a continuous spectrum, not a distinct entity. In the HCP database, agoraphobia was evaluated through the SSAGA interview; however, we did not have access to the complete results of the interview, which examines autonomic and vestibular symptoms associated with avoidance in detail. Thus, our findings may be limited by the extent of symptoms that were available to us to characterize our agoraphobic group.

## Data Availability

Publicly available datasets were analyzed in this study. This data can be found here: http://www.humanconnectome.org/.

## Ethics Statement

Data were provided by the Human Connectome Project, WU-Minn Consortium (Principal Investigators: David Van Essen and Kamil Ugurbil; 1U54MH091657).

## Author Contributions

II and NT managed the overall project (conceptualization and methodology). AC performed graph metric calculations. AC and NT performed statistical analysis. II, AC, and NT prepared the original draft, critically reviewed the manuscript, and approved the final manuscript as submitted. JS, FL, and LP provided methodological perspective on the study, critically reviewed, and edited the initial manuscript and approved the final manuscript as submitted. All authors contributed to manuscript revision and editing.

### Conflict of Interest Statement

The authors declare that the research was conducted in the absence of any commercial or financial relationships that could be construed as a potential conflict of interest.
